# Noncanonical NF-κB in Cancer

**DOI:** 10.3390/biomedicines6020066

**Published:** 2018-06-05

**Authors:** Matthew Tegowski, Albert Baldwin

**Affiliations:** 1 Curriculum of Genetics and Molecular Biology, The University of North Carolina at Chapel Hill, Chapel Hill, NC 27599, USA; tegowski@email.unc.edu; 2 Lineberger Comprehensive Cancer Center, The University of North Carolina at Chapel Hill, Chapel Hill, NC 27599, USA

**Keywords:** noncanonical NF-κB, cancer, cellular signaling, inflammation, tumor initiating cells, NIK, RelB, p52

## Abstract

The NF-κB pathway is a critical regulator of immune responses and is often dysregulated in cancer. Two NF-κB pathways have been described to mediate these responses, the canonical and the noncanonical. While understudied compared to the canonical NF-κB pathway, noncanonical NF-κB and its components have been shown to have effects, usually protumorigenic, in many different cancer types. Here, we review noncanonical NF-κB pathways and discuss its important roles in promoting cancer. We also discuss alternative NF-κB-independent functions of some the components of noncanonical NF-κB signaling. Finally, we discuss important crosstalk between canonical and noncanonical signaling, which blurs the two pathways, indicating that understanding the full picture of NF-κB regulation is critical to deciphering how this broad pathway promotes oncogenesis.

## 1. Introduction

The Nuclear factor kappa-light-chain-enhancer of activated B cells (NF-κB) pathway is an important regulator of innate and adaptive immune responses, where it regulates responses to pathogens, as well as T and B cell activation [1]. NF-κB regulates immune responses by promoting the transcription of proinflammatory and antiapoptotic genes. Additionally, diverse stimuli such as UV radiation, DNA damage, cytokines, growth factors, and reactive oxygen species have all been shown to lead to NF-κB activation [2]. Activation of NF-κB subunits leads to their nuclear translocation and activation of transcription, and the NF-κB pathway is known to regulate the transcription of many genes including proinflammatory cytokines and chemokines (e.g., IL-6 [3]), cell cycle genes (e.g., cyclin D1 [4]), antiapoptotic genes (e.g., bcl-2 [5]), and extracellular proteases (e.g., MMP3 [6]). Chronic inflammation and DNA damage have long been associated with the development of cancer, and dysregulation of the NF-κB pathway has important effects in cancer [7].

**Figure 1:**
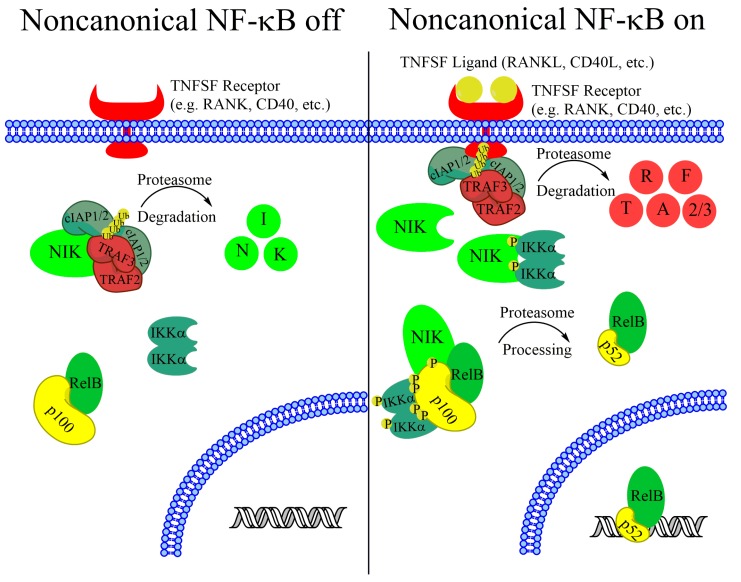
Overview of the Noncanonical NF-κB signaling pathway. In the absence of a stimulus the critical kinase NIK is constitutively targeted for degradation by a ubiquitination complex containing TRAF2, TRAF3, cIAP1, and cIAP2. Upon receptor activation, NIK is stabilized, leading to IKKα activation and p100 processing to p52. RelB-p52 dimers then translocate to the nucleus and activate transcription.

NF-κB pathway activation leads to transcription regulation by dimers of 5 related transcription factors (RelA/p65, RelB, c-Rel, NFKB1/p105, and NFKB2/p100). NFKB1/p105 and NFKB2/p100 subunits require posttranslational proteolytic processing before they can support transcription activation. NFKB1/p105 is thought to be constitutively processed into the active p50 subunit concurrent with translation [8], whereas NFKB2/p100 remains unprocessed until noncanonical pathway activation induces its proteasome-dependent processing into the active p52 subunit (Figure 1) [9]. Although many combinations of dimers have been observed, the most widely studied dimers are the RelA-p50 dimer, which is primarily activated by canonical NF-κB signaling, and the RelB-p52 dimer, which is activated by noncanonical NF-κB signaling.

Activation of canonical NF-κB is dependent on a kinase complex that contains the scaffold protein NF-κB essential modifier (NEMO) and the inhibitor of NF-κB kinase β (IKKβ) and IKKα. Upon activation, IKKβ phosphorylates the inhibitor of NF-κB α (IκBα) that binds to and inhibits RelA-p50 dimers, confining the dimers to the cytoplasm. Phosphorylated IκBα is rapidly targeted for degradation by the proteasome, with subsequent accumulation of nuclear RelA-p50 [10,11]. Noncanonical NF-κB, however, is dependent on the stabilization of a labile kinase, NF-κB-inducing kinase (NIK) and the catalytic activity of IKKα.

While the canonical NF-κB pathway is rapidly inducible and can be activated by inflammatory cytokines and other stimuli, the noncanonical NF-κB pathway is primarily activated by a set of cytokine/receptor pairs in the tumor necrosis factor receptor superfamily including BAFF receptor (BAFFR), CD40, lymphotoxin B receptor (LTβR), Fn14, and receptor activator of nuclear factor kappa-B (RANK) [12,13,14,15,16]. In the absence of a stimulus, noncanonical NF-κB is kept inactive by the continual ubiquitination and proteasomal degradation of the critical upstream kinase NIK. TNF-receptor associated factor 3 (TRAF3) is critical for maintaining low basal NIK levels, and cells with inactivating TRAF3 mutations have upregulated NIK protein levels [17,18,19]. TRAF3, however does not have ubiquitinase activity. Instead, TRAF3 recruits NIK to a degradation complex containing TRAF2 and cellular inhibitors of apoptosis 1 (cIAP1) and cIAP2 [20,21,22]. All components of this complex are required, although cIAP1 and cIAP2 seem to have redundant functions and NIK stabilization does not occur unless both cIAP1 and cIAP2 are reduced [20]. Upon complex formation, NIK protein is marked for proteasome-mediated degradation with K48-linked ubiquitin chains [17]. The continual degradation of this critical kinase in the absence of a stimulus keeps the noncanonical NF-κB pathway inactive.

Upon receptor stimulation, the NIK-degradation complex is recruited to the active receptor complex. Instead of marking NIK for degradation, cIAP1 and cIAP2 ubiquitylate TRAF2 and TRAF3, which are then rapidly degraded (Figure 1) [17,20,23]. Without TRAF3 recruiting NIK for destruction, NIK protein rapidly accumulates. NIK phosphorylates residues on p100 and the downstream kinase IKKα [9,24,25]. Phosphorylation of IKKα activates its kinase activity and IKKα phosphorylates several residues on the C-teminus of p100 [9]. Although NIK phosphorylates p100, IKKα activity is required for p100 processing, and mutating IKKα phosphorylation sites on p100 prevents processing and pathway activation (Figure 1) [9,25]. Phosphorylated p100 is recognized by the E3 ubiquitin ligase βTRCP and processed into the p52 subunit in a proteasome-dependent manner [26]. Although IKKα activity is required for p100 processing, and IKKβ activity is not, it is unclear what the IKKα-containing complex that phosphorylates p100 consists of. Recent work from the Ghosh lab suggests that IKKα can form a hexamer in vitro, and IKKα mutated at residues predicted to be important for hexamerization fails to transduce noncanonical signaling [27]. However, size exclusion chromatography shows that overexpressed IKKα exists predominately as dimers in cells [27]. This suggests that if the hexadimeric complexes exist, they may be transient upon noncanonical receptor stimulation. After processing, the IκB-like inhibitory properties of p100 (see below) are lost and the RelB-p52 dimer can promote transcription of target genes (Figure 1). Although noncanonical and canonical NF-κB regulate different target genes, there is significant overlap in regulated genes [28]. While there is evidence that these differences arise from small variations in the DNA sequence of κB sites [29,30], there is still a lot unknown about how dimer specificity is determined and what the biological relevance may be [28]. In fact, although the noncanonical pathway has been elucidated as quite separate from the canonical NF-κB pathway, there is significant crossregulation between the components of each pathway, emphasizing the importance of the NF-κB system as a single, highly complex system with disease relevance in many types of cancer.

The processing of p100 is a critical event in the noncanonical NF-κB pathway, in order to generate the functional p52 transcription factor. This processing removes C-terminal ankyrin repeats, which are similar to those in the IκB proteins that sequester and inhibit the canonical RelA-p50 dimers. Therefore, p100 binds and inhibits RelB in the absence of an activating signal, and p100 processing is a critical event in the activation of the noncanonical NF-κB pathway [31,32,33]. Further, p100 has been shown to inhibit the canonical pathway by binding and inhibiting RelA, an activity known as IκBδ, through its ankyrin repeats [28,34]. In this review, we explore what is currently known about the impact of noncanonical NF-κB components on cancer initiation, growth, and survival. We also explore functions of noncanonical NF-κB kinases outside of their traditional roles in regulating this pathway. Further, we explore interesting crossregulation mechanisms linking canonical NF-κB components with noncanonical NF-κB.

## 2. Noncanonical NF-κB Activation in Multiple Myeloma

Noncanonical NF-κB is critical for B-cell homeostasis as well as lymph node and germinal center establishment [35,36,37,38,39]. Multiple myeloma (MM) is caused by uncontrolled proliferation of bone marrow plasma cells, which are post germinal center mature B cells that secrete antibodies into the serum [40]. Since both canonical and noncanonical NF-κB are critical for B-cell development and germinal center formation [41], it is perhaps expected that NF-κB activation would be an important development in MM. MM cell adhesion to bone marrow stromal cells induces NF-κB activity and drug resistance [42,43]. Further, production of the NF-κB target gene RANKL in the tumor cells activates osteoclasts, leading to bone resorption at the sites of lesions [16,44].

Although dysregulation of the NF-κB pathway occurs in many tumor types, mutations in NF-κB pathway regulators are rare in solid tumors [7]. However, NF-κB-activating mutations are estimated to occur in about 20% of multiple myeloma patients [45,46]. Mutations in CD40, LTβR, NIK, TRAF2, TRAF3, cIAP1, cIAP2, CYLD, NFKB1/p105, and NFKB2/p100 have been observed, with TRAF3 inactivation being the most frequent alteration. NIK protein is stabilized in TRAF3 mutant MM cell lines, which induces constitutive processing of p100 to p52 and hyperactive noncanonical NF-κB [45,46].

Both canonical and noncanonical NF-κB depend on proteasome activity for activation. Canonical NF-κB requires the degradation of the IκB proteins to release the RelA-p50 dimers, whereas noncanonical NF-κB requires the proteasome for p100 processing to p52. The glucocorticoid dexamethasone, which can block NF-κB signaling [47,48], and the proteasome inhibitor bortezomib are impactful treatments for MM in the clinic [49]. Interestingly, patients with TRAF3 mutations, and therefore hyperactive noncanonical NF-κB, are resistant to dexamethasone treatment. However, TRAF3 mutant tumors were found to be exquisitely responsive to proteasome inhibition with bortezomib [45]. This heightened sensitivity to proteasome inhibition in tumors with hyperactive NF-κB can informs treatment decisions. Further, these lessons may apply to tumor types other than MM where NF-κB is persistently activated, even if no mutations in the pathway are present.

Even though primarily noncanonical-regulating genes are mutated in MM, activation of canonical NF-κB is observed in many MM cells. Further, inhibition of both canonical and noncanonical pathways is effective in reducing growth and promoting cell death in many MM cell lines [46,50], indicating that canonical NF-κB is also critical for cell survival in many MM cells. This suggests that while MM has a special dependence on activation of the noncanonical pathway, significant crosstalk between the NF-κB pathways occurs, and both are often required for MM cell survival and proliferation. Importantly, targeting NF-κB activity by inhibiting IKKs or the proteasome induces cytotoxicity in MM cells [46,49]. Finding new methods of targeting NF-κB in multiple myeloma cells, as well as preventing resistance to these therapies would be important to improving treatment success in this disease.

## 3. Noncanonical NF-κB in Other Cancers

Noncanonical NF-κB has been shown to regulate mammary gland development [51,52]. It is, therefore, intriguing that activity of the noncanonical NF-κB transcription factors RelB and p100/p52 (NFKB2) have been implicated in promoting breast cancer. It has been known for decades that breast cancer cells show increased NF-κB activity, which supports growth and survival [53]. More recently, the expression of RelB and NFKB2 was shown to be elevated in estrogen receptor negative (ER^-^) breast tumors, compared to ER^+^ tumors [54]. ER^−^ tumors are generally more aggressive and have a worse prognosis than ER^+^ tumors. Additionally, estrogen receptor has been shown to repress NF-κB activity [55], and FoxA1, a cofactor critical for estrogen receptor transcriptional activity [56], also directly represses RelB expression [57]. RelB in turn has been shown to inhibit estrogen receptor expression in breast cancer cells by upregulating a transcriptional repressor Blimp1 [58]. RelB expression in breast cancer cells promotes an epithelial-to-mesenchymal transition (EMT), and supports the self-renewal of tumor initiating cells [55,59,60]. Further, patients with higher expression of RelB and NFKB2 had decreased disease free survival as well as decreased overall survival [54]. The noncanonical NF-κB transcription factors are upregulated in ER^-^ breast cancers, and they effect important processes such as EMT and self-renewal. However, since the critical upstream kinase NIK, has not been found to be widely stabilized in breast cancer cells, more work needs to be done to uncover the mechanisms by which RelB and NFKB2 expression and activity are promoted in breast cancers.

The activity of another hormone receptor, androgen receptor (AR), is intimately linked with noncanonical NF-κB signaling. Many prostate tumors are driven by AR activity, and can be effectively treated with surgical or chemical androgen deprivation therapy [61]. Some prostate cancers will acquire resistance and become castration-resistant tumors, often as a result of AR ligand-independent activity [62]. Work from the Gao laboratory demonstrated that p52 expression could support tumor growth of androgen-dependent LNCaP prostate cancer cells in castrated mice [63]. Further, p52 could induce the nuclear localization and DNA binding activity of AR in the absence of ligand. The expression of p52 is high in a castration-resistant subline of LNCaP cells, and importantly, knockdown of p52 in these cells reduces AR activity and DNA binding [63]. How noncanonical NF-κB is activated in castration-resistant prostate cancer needs to be elucidated to determine whether inhibition of noncanonical NF-κB may be a druggable pathway in castration-resistant prostate cancer.

Activation of noncanonical NF-κB, along with NIK stabilization and constitutive p100 processing, has also been observed in pancreatic cancer cell lines [64,65]. Interestingly, multiple mechanisms for noncanonical NF-κB activation in pancreatic cancer cells have been discovered. First, work from the Storz lab has shown that NIK stabilization is critical for proliferation and tumorigenicity of pancreatic cells [66]. Further, expression of a negative regulator of NIK, TRAF2, was shown to be limited as a consequence of constitutive degradation [66]. Work from our lab has also shown that GSK3α activity activates both canonical and noncanonical NF-κB signaling, and that GSK3α activity supports the proliferation and survival of pancreatic cancer cells [67]. In addition to described roles in hematologic malignancies, prostate cancer, breast cancer, and pancreatic cancer, the noncanonical NF-κB pathway and its components have been shown to support the proliferation, survival, or tumor initiating cells of glioma [68,69], ovarian [70], and endometrial cancers [71].

Recently, RelB-p52 dimers were shown to regulate a group of nucleic acid editing enzymes (APOBECs) that are implicated in tumor progression [72]. A growing body of evidence suggests that APOBEC enzymes are responsible for increasing tumorigenesis by inducing mutations at cytosine residues immediately preceded by a thymine [72], especially in breast and ovarian cancers [73]. APOBEC enzymes are a family of enzymes that deaminate cytosines on viral RNA to leave an abundance of uracil residues. This activity behaves in practice as hypermutation of viral RNA [74]. Although they edit RNA residues, APOBEC proteins have been shown to mutate DNA in cancer, where the resulting genomic hypermutation can drive tumor growth. Given its roles in the innate immune response, a possible connection between APOBEC expression and NF-κB activity seems likely. Indeed, the noncanonical NF-κB transcription factors RelB and p52 were recently shown to induce APOBEC3B expression in some cancer cells [75]. Leonard et al. demonstrated that protein kinase C (PKC) activation leads to increased APOBEC3B expression by inducing the binding of RelB and p52 to several sites in the APOBEC3B promoter. Further, this activity was sensitive to both IKK and proteasome inhibitors, indicating that IKK activation and p100 processing are required for this activity [75].

**Figure 2:**
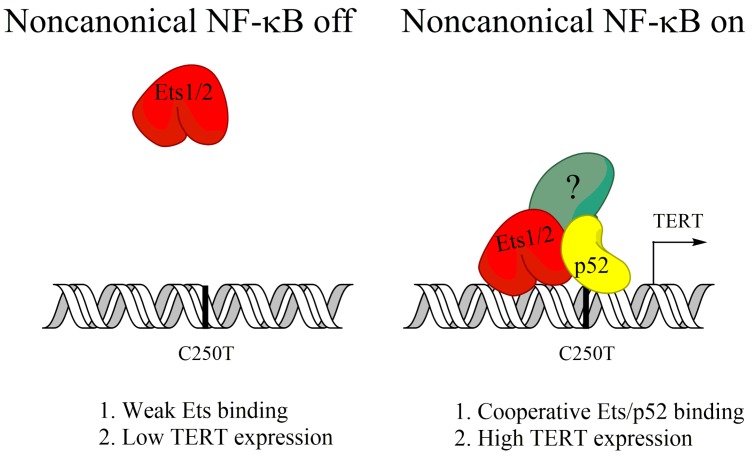
Cooperation of Ets and p52 in promoting TERT expression when promoter is mutated. The C250T mutation in the TERT promoter creates a p52 binding site. When noncanonical NF-κB is activated in C250T mutant cells, p52 can recruit Ets transcription factors to the mutated TERT promoter and drive transcription.

TERT is the catalytic subunit for the telomerase enzyme, which maintains the protective telomeres at the ends of chromosomes that would otherwise be lost progressively every cell division [76]. Maintenance of telomeres is required for the indefinite proliferative potential of cancer cells and TERT activity can be measured in 80–90% of cancers [76,77]. In some cancers, TERT upregulation occurs as a result of point mutations at hotspots in the TERT promoter, which allow for the binding of new transcription factors, such as the Myc or Ets transcription factors [78,79]. Activation of noncanonical NF-κB, but not canonical NF-κB, was shown to increase TERT expression in glioblastoma cells containing one of these hotspot mutations (C250T) [80]. Ets1/2 transcription factors were found to be recruited to the mutated TERT promoter by p52 in order to induce TERT expression (Figure 2), and reversion of the C250T promoter mutation blocked p52 binding, and noncanonical NF-κB-induced TERT expression [80]. Further, noncanonical NF-κB was required for in vivo tumor growth [80]. This shows that activation of noncanonical NF-κB can support tumor growth of some glioblastoma by upregulating TERT expression.

Although p52 has generally been shown to promote noncanonical NF-κB and oncogenic functions, its precursor, p100, has been demonstrated to act as a tumor suppressor in an NF-κB-independent mechanism [81]. p100, but not p52, was shown to inhibit anchorage-independent growth by directly interacting with ERK2, and via inhibition of c-Jun/AP-1, inducing the downregulation of miR-494. PTEN expression, normally inhibited by microRNA (miRNA) miR-494, increased [81]. Another study from the same group demonstrated that p100, but not p52, could limit the proliferation of bladder cancer cells by promoting miR-302 production via CREB activity. Additionally, miR-302 was demonstrated to suppress cyclin D1 expression, leading to decreased proliferation [82].

The activity of RelB and NFKB2/p52 has been demonstrated in numerous cancer types, and they regulate a diverse set of genes. When activated, these noncanonical NF-κB transcription factors promote tumor initiation, growth, and survival. Inhibition of their activity could yield advances in the treatment of numerous cancers, including breast, brain, ovarian, and prostate.

## 4. RelB-p52 and EZH2 Cooperation in Cancer

**Figure 3:**
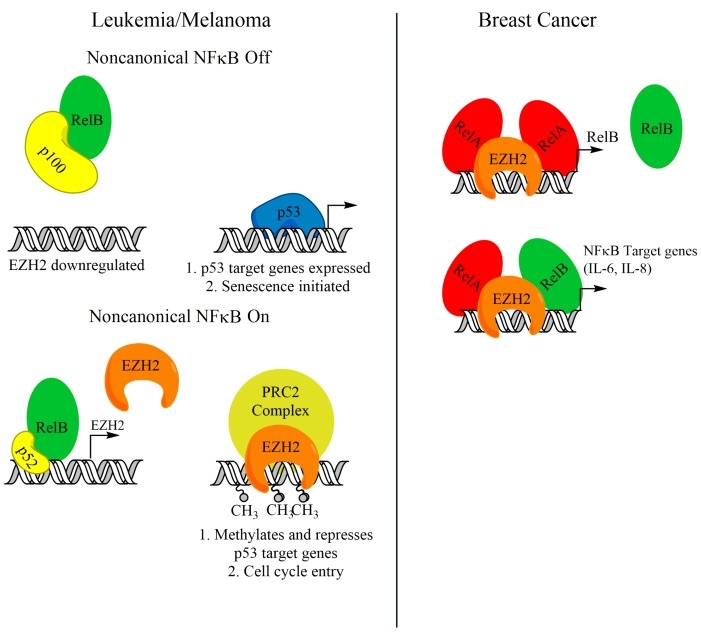
Crosstalk between EZH2 and noncanonical NF-κB components in CLL, melanoma, and breast cancer. Work in CLL and melanoma shows that RelB-p52 dimers can promote the transcription of EZH2. EZH2 can then promote a senescence bypass by repressing p53 target genes.

As discussed earlier, activation of the noncanonical NF-κB transcription factors, namely RelB and p52, can support the growth of several cancer types. Recently, a body of evidence has emerged linking RelB-p52 activity with enhancer of zeste homolog 2 (EZH2). EZH2 is a histone methyltransferase that, as the catalytic subunit of the polycomb repressive complex 2 (PRC2), trimethylates H3K27 (H3K27me3) and leads to gene repression [83,84,85,86]. EZH2, through its catalytic activity, has been shown to be a tumor promoter, because it can repress tumor suppressors such as p16^Ink4a^ and p14^ARF^ [87]. Iannetti et al. showed that noncanonical NF-κB promotes the expression of EZH2 in chronic lymphocytic leukemia (CLL) cells. Active RelB-p52 promoting EZH2 expression supports a blockade on p53-mediated entry into senescence by downregulating p53 target genes (Figure 3). ChIP analysis in human fibroblasts revealed that RelB and p52 bind to the EZH2 promoter [88], indicating direct transcriptional regulation of EZH2 by noncanonical NF-κB. A supporting study in melanoma cells suggests that noncanonical NF-κB upregulation of EZH2 may be a general mechanism to bypass p53-induced senescence [89]. Interestingly, this study showed that NIK protein was stabilized in melanoma cells and is critical for EZH2 expression. Canonical NF-κB subunits were not found at the EZH2 promoter, and knockdown of RelA had no effect on EZH2 expression [89]. This suggests that activation of noncanonical NF-κB can support EZH2 expression and avoid senescence, without significant crosstalk with canonical NF-κB pathway members.

Although EZH2 is a well-established transcriptional repressor, functioning through its catalytic activity to methylate H3K27, a growing body of evidence has shown that EZH2 has PRC2-independent roles as a transcriptional activator. SWI/SNF mutant tumors were shown to require EZH2 for growth. Interestingly, EZH2 depletion in some SWI/SNF mutant tumors was rescued with a catalytically inactive mutant, suggesting alternative functions of EZH2 [90]. Further, EZH2 was found to act as a coactivator to support the transcriptional activation of target genes controlled by Notch [91], Wnt [92,93], estrogen receptor [92], and androgen receptor [94]. Interestingly, EZH2 was also found to support NF-κB target gene expression in triple-negative breast cancer cells by acting as a coactivator in a complex containing RelA and RelB (Figure 3) [95]. This was further supported by a study from our group, which showed that EZH2 and RelA localized to the RelB promoter and supported transcription of RelB in triple-negative breast cancer cells (Figure 3). This activity was critical for the cancer cells to maintain tumor initiating cells [59]. Intriguingly, this NF-κB-supporting activity in triple-negative breast cancer cells is independent of its methyltransferase activity, and PRC2 complex members, like SUZ12 [60,95]. Since EZH2 enzymatic activity is dispensable for this regulatory mechanism, more work needs to be done to elucidate therapeutic mechanisms to target this noncanonical function of EZH2.

## 5. Noncanonical IκB Proteins

Originally discovered to inhibit the NF-κB response, IκB proteins are generally considered to be cytoplasmic molecules that bind and inhibit the transcriptionally active RelA/p65 subunits. However, there are several nontraditional IκB proteins that reside in the nucleus and generally act as transcriptional activators. Two of the most well-studied nuclear IκBs are Bcl-3 and IκBζ. Bcl-3 contains several ankyrin repeat domains, like all other IκB proteins. Unlike other IκB proteins, Bcl-3 preferentially binds p50 and p52, rather than the transcriptionally active RelA, RelB, or c-Rel [96,97]. Since Bcl-3 was originally shown to inhibit the DNA binding of p50 homodimers, this led to the hypothesis that Bcl-3 inhibits NF-κB activity [98]. Further Bcl-3 can blunt the NF-κB-dependent expression of TNFα in response to LPS stimulation [99]. Bcl-3 has also been shown to recruit repressors to viral genes and repress transcription [100]; however Bcl-3 has generally been observed to promote transcriptional activation. Additionally, Bcl-3 expression is induced by NF-κB activity [101]. In response to NF-κB activation, Bcl-3 binds p50 and p52 and acts as a transcriptional activator, promoting the transcription of anti-apoptotic and proliferation genes, such as cyclin D1 [102,103,104,105]. In this regard, Bcl-3 has also been shown to inhibit p53 by promoting the transcription of its negative regulator, hdm2 in breast cancer cells [104]. Completing the regulatory circle, p53 has been shown to decrease cyclin D1 by inhibiting Bcl-3. In the absence of Bcl-3, p52 associates with HDACs at the cyclin D1 promoter, inhibiting transcription [106]. Increased expression of Bcl-3 was observed in breast cancer [103], skin squamous cell carcinoma [107], endometrial cancer [108], nasopharyngeal cancer [109], and some lymphomas [110]. Although established as an oncogene, there are still many questions as to how Bcl-3 activity is regulated. It has been known to be modulated by phosphorylation nearly since its discovery [96], and both IKKα and IKKβ have been shown to rapidly phosphorylate Bcl-3 and stimulate Bcl-3-p50 complexes to initiate transcription [111]. Recently, work from the Ghosh lab has given a glimpse into the complex regulation of Bcl-3 by phosphorylation. They showed that Akt phosphorylates Bcl-3 to increase its stabilization and nuclear localization, while IKKs and ERK phosphorylates Bcl-3 to promote its transcriptional activity [112]. Still, there is much that is unknown as to how the oncogenic functions of Bcl-3 are regulated.

Another nuclear IκB protein, IκBζ, promotes the transcription of inflammation-associated genes like IL-6 [113,114]. In general, IκBζ mRNA expression is kept silent and its mRNA is unstable. Upon stimulation of toll-like receptors (TLRs) or IL-1β, but not TNFα, IκBζ mRNA is stabilized and its transcription is initiated in an NF-κB-dependent manner [115,116]. IκBζ is both a target gene of NF-κB, and a coactivator, as it is critical to proper NF-κB target gene expression downstream of TLRs and IL-1β [113,117,118]. Like Bcl-3, IκBζ preferentially interacts with p50 and p52, but not RelA [119,120]. p50 homodimers, which are normally considered to be transcriptionally inactive, activate the transcription of NF-κB target genes when associated with IκBζ [118]. Recently, Nogai and colleagues showed that IκBζ expression is critical for activated B cell-like diffuse large cell B cell lymphoma (ABC-DLBCL) cells, but not germinal cell B cell-like DLBCL (GC-DLBCL) or multiple myeloma cells. Not only did they show that IκBζ expression is critical to maintain the expression of several NF-κB target genes, but that knockdown of IκBζ caused cytotoxicity in ABC-DLBCL cells, emphasizing its important roles in ABC-DLBCL [120]. Often overlooked in NF-κB research, these nontraditional IκB transcription factors have important roles in supporting NF-κB responses in immunity and disease and deserve more research.

## 6. Alternative Functions of the Noncanonical Kinases

### 6.1. Other Functions of IKKα

As the critical kinase in mediating activation of the canonical NF-κB pathway, IKKβ has been more highly studied than IKKα. IKKα does not play a major role in controlling IκBα degradation in response to TNFα [121,122]. However, IKKα does play a role in regulating NF-κB target gene expression [123,124,125]. Interesting evidence that IKKα may shuttle into the nucleus provided evidence of potential alternative functions [126]. Further clarification came when IKKα was found to phosphorylate serine 10 on histone 3 (H3S10), and loss of IKKα caused defects in NF-κB-dependent transcription [127,128]. Much of this mechanistic work was done in MEFs and did not establish whether nuclear IKKα has any consequences in cancer.

**Figure 4:**
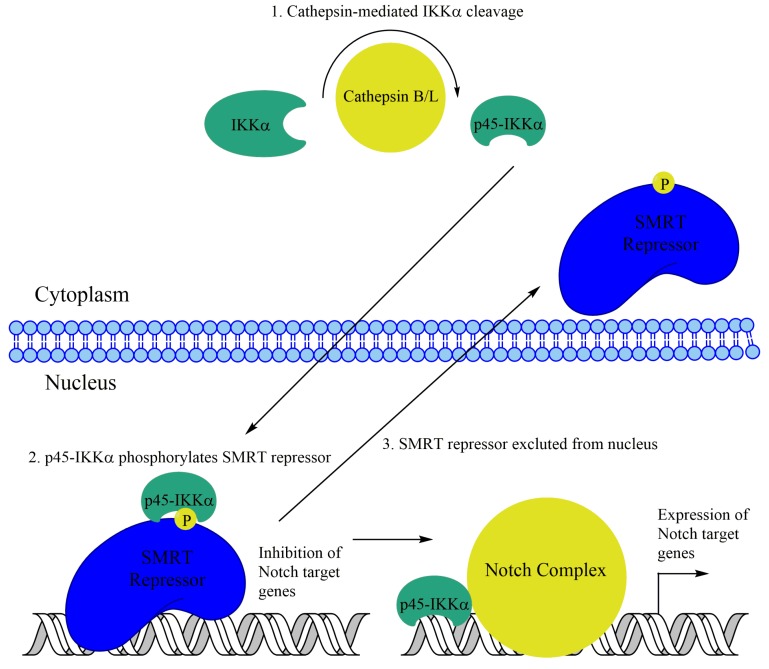
Nuclear IKKα promotes the expression of Notch target genes in colorectal cancer cells. A truncated p45-IKKα has been shown to phosphorylate the SMRT repressor, leading to its exclusion from the nucleus, leading to the expression of Notch target genes.

Subsequently, nuclear IKKα was observed in a prostate cancer cell line. IKKα, but not IKKβ, was found to phosphorylate the SMRT corepressor in the prostate cancer cell line DU145 in response to laminin attachment. This phosphorylation leads to SMRT and the associated repressor HDAC3 to be exported from the nucleus, leading to optimal activation of NF-κB target genes like cIAP1/2 and IL-8. This mechanism appears to be independent of IKKα-mediated H3S10 phosphorylation [129]. Additionally, interesting work in colorectal cancer cells has implicated IKKα-mediated phosphorylation of SMRT with changes in NF-κB-independent transcription. Nuclear IKKα and phospho-SMRT were observed in colon cancer cell lines as well as patient samples. Upon further investigation, IKKα was observed to phosphorylate SMRT, leading to its exclusion from the nucleus, similar to the prostate cancer cells. Additionally, IKKα presence at Notch target genes in colon cancer cells was identified using ChIP analysis (Figure 4). Further, IKKα was shown to promote Notch-dependent transcription and tumorigenesis in colon cancer [130]. More recently, a truncated p45-IKKα was observed in colorectal cancer cells. This fragment, generated by cathepsin protease activity, was shown to be critical for SMRT phosphorylation as well as expression of cIAP1/2 and several Notch target genes (Figure 4) [131].

Additionally, IKKα has also been shown to suppress gene expression [132]. IKKα activity was found to be associated with decreased expression of a metastasis-associated gene Maspin. Further, this is dependent on both IKKα kinase activity and nuclear localization, but it does not require IKKβ activity [132]. This fascinating result has potentially exciting consequences, as targeting this IKKα activity in prostate cancer with small molecule inhibitors could reduce metastatic burden. However, the mechanism by which IKKα mediates gene repression is not known, and IKKα has not yet been reported to bind at the Maspin locus using ChIP. It is possible these results indicate that activation of noncanonical NF-κB mediated by IKKα could be repressing gene expression as shown in multiple myeloma [133,134,135]. These mechanisms show that in addition to acting as a primary mediator for noncanonical NF-κB pathway activation, nuclear IKKα directly regulates gene transcription through the regulation of histones and chromatin-associated factors.

### 6.2. Alternative Roles for NIK

NIK is almost exclusively studied for its critical role in promoting noncanonical NF-κB. As described earlier, NIK is expressed at low levels due to constitutive degradation until a stimulus induces its stabilization, and as a consequence, NIK is not stabilized in many cell types. Nonetheless, intriguing functions for NIK have been uncovered. Birbach et al. showed that NIK has a nucleolar targeting sequence that is required for nuclear localization [136]. Interestingly, they show that a mutant of NIK that preferentially accumulates in the nucleolus is less efficient at inducing noncanonical NF-κB activity, suggesting that nucleolar NIK is either less efficient at activating NF-κB, or more interestingly, that it has alternative functions there. Further, nuclear localized NIK can be observed by immunofluorescence in the triple-negative breast cancer cell line MDA-MB-231 [136]. This interesting finding further suggests that nuclear localized NIK may have functions in cancer cells. Several other triple-negative breast cancer cells have been shown to have stabilized NIK, and NIK may promote breast cancer stemness [137,138]. The potential impact of NIK stabilization has not been thoroughly studied in breast cancer. Since EZH2 promotes RelB production and tumor initiating cells in triple-negative breast cancer [60], it is interesting to consider what effects NIK stabilization may have on EZH2-RelB crossregulation.

As discussed earlier, several reports indicate that NIK stabilization and subsequent noncanonical NF-κB activation generally supports the growth and survival of tumor cells. However, a recent report unexpectedly shows that NIK stabilization in certain contexts can act as a tumor suppressor. Canonical NF-κB activity is increased in AML, and it supports survival [139]. Xiu et al. show that NIK stabilization in AML cells induces noncanonical NF-κB, but nuclear RelA was reduced, which led to decreased tumor growth [140]. Interestingly, RelA overexpression promoted tumor growth, whereas RelB overexpression decreased tumor growth [140]. This has interesting implications on NF-κB biology. As described below there is significant crosstalk between canonical and noncanonical NF-κB, and this study suggests that in AML, there is an antagonistic relationship between canonical and noncanonical NF-κB, and activation of canonical NF-κB is ultimately critical to tumor survival.

NIK has many interesting functions due to its role as a noncanonical NF-κB activator. However, its activity has also been shown to regulate processes completely independent of NF-κB activity. Recently, Jung et al. have shown that NIK is localized to mitochondria in a glioma cell line [141]. Not only is NIK localized to mitochondria, but it promotes mitochondrial division and invasion. NIK does this by promoting the mitochondrial localization of Drp1, a GTPase required for mitochondrial fission. Interestingly, this activity is not dependent on NF-κB or IKKα, but can be stimulated by the TNF related ligand TWEAK [141]. TWEAK is a known noncanonical NF-κB inducing agent that can stimulate NIK and IKKα activity [15]. It is interesting that TWEAK induces NIK to support mitochondrial fission, but not NF-κB activation [141]. This intriguing study shows that NIK has alternative functions in some cancer cells that have important effects on metabolism and invasion. Interestingly, Drp1 activity has also been found to support glioblastoma tumor initiating cells [142] and pancreatic tumor growth in vivo [143], highlighting the potential impact of targeting this pathway. NIK is an understudied kinase that has complex effects on development and cancer, and more work needs to be done to identify potential targeting agents against this interesting kinase.

## 7. Crosstalk between Canonical and Noncanonical NF-κB

**Figure 5:**
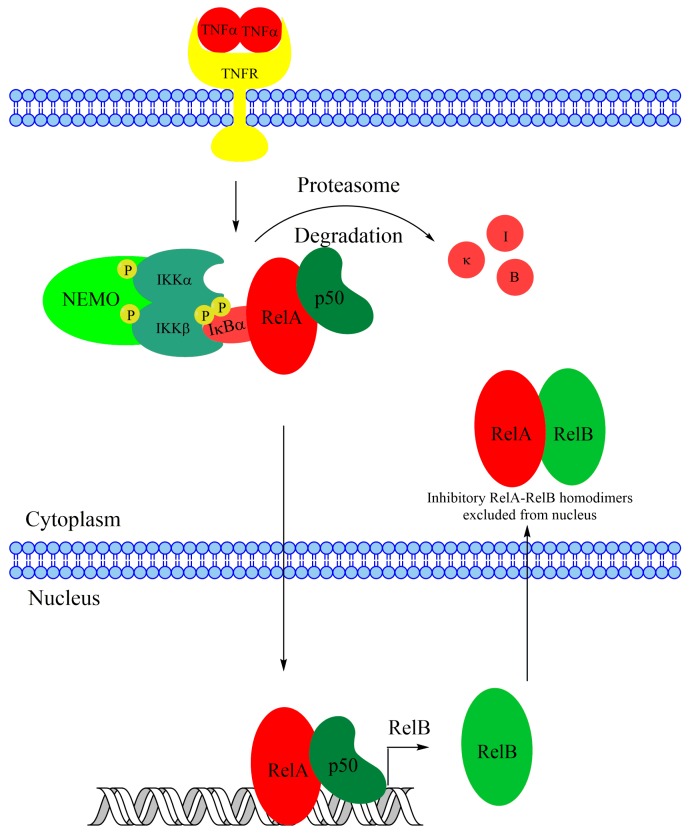
Canonical NF-κB activity stimulates RelB expression. Activation of RelA-p50 dimers with TNFα induces increased expression of RelB. RelB induction serves as negative feedback by binding RelA and sequestering it in the cytoplasm.

The NF-κB pathway involves numerous regulators and co-regulators, many of which have additional known functions outside of NF-κB regulation. Additionally, NF-κB activation is achieved by a variety of stumuli, and the induced target genes can vary by stimulus and cell type, thus the NF-κB response exhibits significant complexity. The noncanonical and canonical NF-κB pathways do not function in isolation, instead, co-regulation and crosstalk are abundant. Importantly, stimulation of canonical NF-κB with TNFα or LPS leads to rapid nuclear accumulation of canonical RelA-p50 dimers, but not RelB [144]. However, increased transcription of RelB in response to TNFα or LPS is dependent on RelA (Figure 5), and this leads to RelB-dependent changes in gene expression, which requires synthesis of new RelB protein [144]. Therefore, canonical NF-κB activation can promote noncanonical target gene expression via induction of RelB transcription. However, RelB has also been shown to suppress RelA DNA binding activity and sequester RelA in the cytoplasm in response to canonical stimuli such as TNFα (Figure 5) [145,146,147], potentially helping to blunt the strong inflammatory response of the canonical NF-κB pathway. Interestingly, RelA-RelB heterodimerization is dependent on contacts in the rel homology domains of each protein, and the interaction is promoted by phosphorylation of S276 on RelA, but inhibited by phosphorylation of RelB on S368 [148,149]. This intriguing antagonism is cell type dependent as the effect was observed in fibroblasts but not in macrophages [145]. Additionally, the RelA-RelB heterodimer also inhibits RelB-mediated transcription after TNFα treatment [148]. The antagonistic relationship between RelA and RelB also has disease relevance, as was explored earlier in a discussion on acute myeloid leukemia. Canonical upregulation of noncanonical NF-κB subunits followed by mutual antagonism between RelA and RelB introduces multiple layers of complexity to the NF-κB response in cells and the regulation of the RelA-RelB heterodimer as well as the S276 residue on RelA needs to be further elucidated to unravel how the NF-κB response is regulated in different cell types.

There is an additional system for inhibition of NF-κB mediated by the full-length precursor molecules p105 (termed IκBγ) and p100 (termed IκBδ). These molecules were observed to form high molecular weight complexes (IκBsomes) that can bind RelA [31,150]. Further, stimulation of canonical NF-κB can induce the expression of p100, acting as a negative feedback mechanism [151,152]. Upon LPS stimulation, IκBsomes dissociated transiently before eventually reforming [150], supporting the hypothesis that IκBsomes can act as a mechanism to limit canonical NF-κB activation. Since the IκBsome inhibitory complexes were found to exist in resting cells, they may also function as reservoirs that can activate both canonical and noncanonical pathway simultaneously upon stimulation. The existence and function of IκBsomes indicates that the NF-κB pathway is complex and that canonical and noncanonical components contribute to the NF-κB response.

As mentioned previously, RelA promotes RelB activity upon stimulation with TNFα. Work from the Baud laboratory showed that RelB activity downstream of TNFα stimulation is mediated through the canonical IKK complex containing IKKα, IKKβ, and NEMO, which phosphorylates RelB after TNFα treatment at S476 [6]. Phosphorylation at S472 dissociates RelB-containing complexes from IκBα, leading to NF-κB activation. Interestingly, phosphorylation at S472 promotes migration in fibroblasts by inducing a RelB-mediated upregulation of matrix metalloprotease 3 (MMP3), indicating an important biological consequence of this pathway [6,153].

The distinction between the two NF-κB pathways is blurred further when the array of subunit dimers that have been discovered is considered. There are 15 possible NF-κB dimer combinations, and 12 of them have been identified [154,155]. Supershift analysis of electrophoretic mobility shift assays (EMSA) provided evidence that alternative dimer combinations occur, even in response to canonical stimuli like TNF [156]. Additionally, stimulation of noncanonical-activating receptors like LTβR induces DNA-binding activity of both canonical RelA-p50 dimers and noncanonical RelB-p52 dimers [157]. The canonical RelA-p50 and noncanonical RelB-p52 dimers are probably the most commonly observed because of binding stability [154]. In particular, structural analysis of the RelA-p50 dimer has shown contacts create a highly stable dimer [154,158]. On the other hand, the RelB dimerization domain differs from that of RelA, leading it to form more stable complexes with both p50 and p52 (see below) [159]. RelA, however, is generally not observed to interact with p52, although it has been seen in RelB-deficient cells [160]. While the RelA-p50 and RelB-p52 heterodimers appear to be the most abundant within cells, other important dimer combinations have been studied.

One of the most well-studied “alternative” dimers is the RelB-p50 dimer, in which the noncanonical RelB molecule binds with the canonical p50. The DNA-binding activity of the RelB-p50 dimer was observed in response to viral proteins [161], bacterial products (LPS) [162], and cytokines [163,164]. The observed activity of the RelB-p50 dimers is usually delayed, being observed hours after a canonical stimulus. However, in dendritic cells, which have high levels of RelB activity, RelB-p50 dimers were found to respond rapidly to canonical stimuli, such as LPS [165]. Interestingly, although RelB is not usually bound to IκB proteins because of the inhibitory action of p100, low levels of p100 in dendritic cells led to RelB association with p50 as well as to IκB proteins. These studies highlight the complexity of the NF-κB pathway, indicating that the two NF-κB pathways are not district entities, but rather two branches of the same pathway with many nodes of cross-regulation.

## 8. Concluding Remarks

Activation of the noncanonical NF-κB pathway has important effects in tissue development as well as disease. Multiple myeloma in particular has a strong dependence on noncanonical NF-κB activation. Activation of noncanonical pathway components such as NIK, RelB, or IKKα have been shown to support tumor progression in various cancers, such as multiple myeloma, DLBCL, glioblastoma, breast, prostate, ovarian, and colon cancers, and inhibitors of NIK or IKKα could have therapeutic benefit for patients with these tumors. While much of the NF-κB-related research focuses on IKKβ/RelA, the noncanonical NF-κB pathway has proven to be equally critical in cancer and other diseases. More work needs to be done to elucidate how canonical and noncanonical NF-κB work to drive tumorigenesis, and determine if they can be effectively targeted.
